# Cholesterol-Lowering Activity of *Lactiplantibacillus pentosus* KS6I1 in High-Cholesterol Diet-Induced Hypercholesterolemic Mice

**DOI:** 10.4014/jmb.2409.04029

**Published:** 2024-11-21

**Authors:** Karthiyaini Damodharan, Sasikumar Arunachalam Palaniyandi, Seung Hwan Yang, Seetharaman Balaji

**Affiliations:** 1 Department of Biotechnology, V.V.Vanniaperumal College for Women, Virudhunagar-626001, Tamil Nadu, India; 2 Department of Biotechnology, Mepco Schlenk Engineering College, Mepco Nagar, Mepco Engineering College Post-626005, Sivakasi, Tamilnadu, India; 3 Department of Biotechnology, Chonnam National University, Yeosu, Chonnam 59626, Republic of Korea.; 4 Department of Biotechnology, Manipal Institute of Technology, Manipal Academy of Higher Education, Manipal- 576104, Karnataka, India

**Keywords:** Probiotics, cholesterol-lowering activity, *Lactiplantibacillus pentosus*, bile salt deconjugation

## Abstract

Hypercholesterolemia is a risk factor of coronary heart disease and cholesterol-lowering probiotics are seen as alternative to drugs for the management of this condition. In the present study, we evaluated the cholesterol-lowering activity of *Lactiplantibacillus pentosus* KS6I1 in high-cholesterol diet-induced hypercholesterolemic mice. The mice were fed with high-cholesterol diet (HCD) and were divided into three groups: HCD group, KS6I1 group (fed with HCD + 200 μl of 10^10^ CFU/ml *L. pentosus* KS6I1), and *L.ac* group (fed with HCD + 200 μl of 10^10^ CFU/ml *L. acidophilus* ATCC 43121 as the positive control). Simultaneously, a normal control diet (NCD) group was maintained. After 6 weeks, the low-density lipoprotein (LDL)-cholesterol and total cholesterol levels were significantly reduced in the blood plasma of KS6I1 group mice. Analysis of liver tissue showed a decrease in total cholesterol and LDL-cholesterol and increase in triglyceride levels in KS6I1 compared to those in HCD group. Fecal total cholesterol and total bile acid content was significantly increased in the KS6I1 group than in other groups. Additionally, gene expression analysis showed that *LDLR*, *SREBF2*, *CYP7A1* genes were significantly upregulated in KS6I1 group compared to the HCD group, while the expression of *NPC1L1* gene was significantly reduced in KS6I1 group compared to HCD group. These observations show that the cholesterol-lowering effect of *L. pentosus* KS6I1 could be attributed to increased excretion of bile acids and cholesterol in the feces of mice. These results indicate that *L. pentosus* KS6I1 could be developed into a potential probiotic for hypercholesterolemia.

## Introduction

*Lactiplantibacillus pentosus* (Basonym: *Lactobacillus pentosus*) belongs to the *plantarum*-group lactobacilli and has been isolated from several fermented foods such as fermenting olives, fermented mulberry leaf powders, fermented teas, glutinous rice dough, corn noodles, chilli sauce, mustard pickles, stinky tofu, dairy products, mustard pickle, fermented idli batter, tempoyak, and sourdoughs [1]. The strains of *L. pentosus* were also isolated from other sources such as corn silage, sewage, human vagina, human stools, etc. [1] and human milk [2].

*L. pentosus* strains have been reported to have a number of potential probiotic properties, which include ameliorating ulcerative colitis in experimental mice [3] and has been shown to increases the abundance of *Akkermansia* [4], inhibition of lipid accumulation in *Caenorhabditis elegans* [5], induces IL-10 producing Tr1 cells and modulates immune response [6], anti-stress and amelioration of stress induced immunosuppression in mice [7], inhibit psoriasis-like skin lesions [8], inhibit multi-drug resistant *Helicobacter pylori* [9], anti-adhesion activity against *E. coli* and *Salmonella typhimurium* [2], inhibits *Candida* infection and prevents against gastric inflammation [10], inhibits *Streptococcus mutans* biofilm formation [11], ameliorates age-dependent memory impairment [12] and age-dependent colitis [13] in rats. Additionally, heat killed *L. pentosus* cells were shown to protect against influenza A virus infection in mouse model. In addition, *L. pentosus* strains have also been shown to produce a number of bacteriocins such as pentocins [14-18], lacidophilin [19], and plantaricin [20].

Whole genome sequence analysis of *L. pentosus* showed the presence of several genes involved in bacteriocin and exopolysaccharide (EPS) production and several genes encoding proteins involved in cell adhesion [20-23].

Apart from these potential probiotic characters, *L. pentosus* has been an important microbe as a starter culture and is widely used for the fermentation of olives, where it has been shown to form biofilms on the surface of olive skin [24, 25] and also reported use as starter in fermented sausage production [26]. In addition, *L. pentosus* strains were also used for the biotransformation of ginsenosides [27] and flavonoids [28].

Cholesterol-lowering activity is one of the most sought-after probiotic characters, since hypercholesterolemia is a major risk factor for cardiovascular diseases, which is a leading cause of death [29, 30]. By the year 2030, about 23.6 million people around the world will be affected by cardiovascular disease [31]. Oral administration of probiotics has been shown to significantly reduce cholesterol levels by as much as 22 to 33% [32]. Administration of probiotic lactic acid bacteria such as, *L. plantarum* PH04 [33], *Lactobacillus reuteri* CRL 1098 [34, 35] and probiotic mixtures [36-38] were shown to reduce serum total cholesterol levels in mice and rat models. Whereas, *L. plantarum* KCTC3928 [39], *L. fermentum* SM-7 [40], *Lactobacillus gasseri* SBT0270 [41], *Bifidobacterium longum* SPM1207 [42, 43] strains were shown to reduce serum total cholesterol and LDL-cholesterol levels in mice and rat. Some strains specifically increase the ratio of HDL-C to LDL-C such as the strain *L. reuteri* CRL 1098 increase the ratio by 17% [35]. Few studies have demonstrated the cholesterol-lowering activity of *L. pentosus* strains in vitro [44, 45] and in vivo [30, 46]. Butter prepared by using *L. pentosus* IBRC-M11043 as adjunct starter culture was observed to have low amount of saturated fatty acids, high amount of unsaturated fatty acids and low levels of cholesterol [44]. Furthermore, heat-killed *L. pentosus* strain S-PT84 was reported to alleviate postprandial hypertriacylglycerolemia by delaying triacylglycerol absorption in the intestine by inhibition of pancreatic lipase in rats [46]. Probiotic Lactobacilli have been reported to reduce cholesterol via several mechanisms which include bile salt hydrolase (BSH) activity in the intestine, cholesterol-assimilation by the bacteria, cholesterol-binding to bacterial cell walls, and/or physiological actions as a result of production of short chain fatty acids [32].

In our previous study, we reported a bile salt hydrolase positive *L. pentosus* strain KS6I1 with in vitro cholesterol assimilation activity [47]. In this study, we report the in vivo cholesterol-lowering activity of *L. pentosus* strain KS6I1 in mice fed with high-cholesterol diet (HCD) and the possible mechanism by which it lowers serum cholesterol.

## Materials and Methods

### Microbial Strain and Culture Conditions

*Lactiplantibacillus pentosus* KS6I1 was from our previous study [47], which has been isolated from fermented radish and was reported to have in vitro cholesterol assimilation activity and bile salt hydrolase activity [47]. The strain KS6I1 was routinely cultured on MRS medium and maintained at 37°C. For animal experiments, the strain was cultured in MRS broth for 24 h and then the bacterial cells were collected by centrifugation at 4000 g for 15 min. The cells were washed with sterile distilled water twice by repeated resuspension and centrifugation. Finally, the KS6I1 cells were resuspended in sterile distilled water to the desired CFU/mL for animal experiments.

### Study of the Effect of *L. pentosus* KS6I1 Feeding on Cholesterol Level in Mice Experimental Animals

ICR mice (5 weeks old male, 35-40 g) obtained from Orient Bio (Republic of Korea) are maintained in cages under a 12-h light/dark cycle at 23 ± 3°C temperature and 40 ± 6% humidity according to our previous study [48]. A high cholesterol diet (40 kcal % fat, 1.25% cholesterol, and 0.5% cholic acid; Cat #101556; Research Diets, USA) was fed to mice for 4 weeks. At the end of 4^th^ week, the high cholesterol diet-fed mice were assigned to three experimental groups: (1) HCD group – this group contained mice fed with high cholesterol diet for 6 weeks; (2) KS6I1 group – this group contained mice fed with high cholesterol diet + 200 μl of 10^10^ CFU/ml *L. pentosus* KS6I1 for 6 weeks; and (3) *L.ac* group – this group contained mice fed with high cholesterol diet + 200 μl of 10^10^ CFU/ml of positive control *Lactobacillus acidophilus* ATCC 43121 for 6 weeks. Additionally, a separate group of mice was fed with normal control diet (Normal) during the entire study period. Groups receiving lactobacilli (KS6I1 and *L.ac*) were orally administered with the respective bacterial cells in distilled water once every day for 6 weeks and only sterile distilled water was administered to the control groups (HCD and Normal). Each group consisted of 6 mice. The body weight of the mice was measured once per week during the entire study period. The food intake (g/mouse/day) was calculated as described previously [31]. The animal experiments were approved by the Committee of Animal Care and Experiment of Chonnam National University (Republic of Korea) with the reference number (CNU-00056) and the mice were maintained in accordance with standard guidelines.

### Tissue Collection and Plasma Lipid Analysis

Collection of tissue samples and analysis of plasma lipids were performed as described in our previous study [31]. Briefly, the mice were sacrificed at the end of the study and the liver and intestine were removed immediately, rinsed, and weighed. Blood samples were collected from the abdominal aorta after making a longitudinal incision in the abdomen to the xiphoid. Plasma was collected from blood samples by centrifugation at 18,000 ×*g* for 10 min and was stored at −80°C until further analysis. Determination of plasma and fecal total cholesterol (T-CHO), triglycerides (TGs), and HDL-cholesterol are by using a commercial kit from Asan Pharm (Republic of Korea). Friedewald formula was used to calculate the levels of LDL-cholesterol (LDLC = TC - HDLC - (TG/5)) [49]. Fecal total bile acid content was determined using a fluorometric assay kit from Cell Biolabs, Inc (Catalog Number STA-631), following the manufacturer’s protocol.

### Analysis of Gene Expression Related to Cholesterol Metabolism in Mouse Liver

Total RNA from mouse liver was extracted using Qiagen RNeasy mini kit (Qiagen Korea, Republic of Korea), following the manufacturer’s protocol. One microgram of RNA was used for cDNA synthesis using PrimeScript II cDNA synthesis kit (TaKaRa, Japan). Amplification of cDNA was performed in Roche LightCycler 96 System (Roche Life Science, USA) using SYBR Premix ExTaq II (Tli RNaseH Plus; TaKaRa Korea Biomedical Inc., Republic of Korea). Real-time PCR was performed according to the protocol reported in our previous study [50]. Amplification of mouse genes *LDLR* (Low density lipoprotein receptor), *SREBF2* (Sterol-regulatory element binding factor 2), *CYP7A1* (cholesterol 7-alpha-hydroxylase), and *ACTB* (β-actin) were according to our previous study [50]. Amplification of mouse *NPC1L1* (Niemann-Pick C1-Like 1) gene was using the forward ATCCTCATCCTGGGCTTTGC and reverse GCAAGGTGATCAGGAGGTTGA primers [51]. The *ACTB* (β-actin) gene served as internal control, and the relative gene expression was calculated using the comparative CT method [52] and the expression of genes was measured as fold change relative to the HCD group.

### Statistical Analysis

Results are expressed as mean ± SD values of three independent experiments. The results of the animal experiments were analyzed using one-way ANOVA followed by Tukey’s HSD test using Origin software (OriginLab Corporation, USA). *P* value < 0.05 was considered statistically significant.

## Results

### Effect of KS6I1 Feeding on Bodyweight Gain, Food Intake and Lipid Content in Blood Plasma of HCD-Fed Mice

**Fig. 1:**
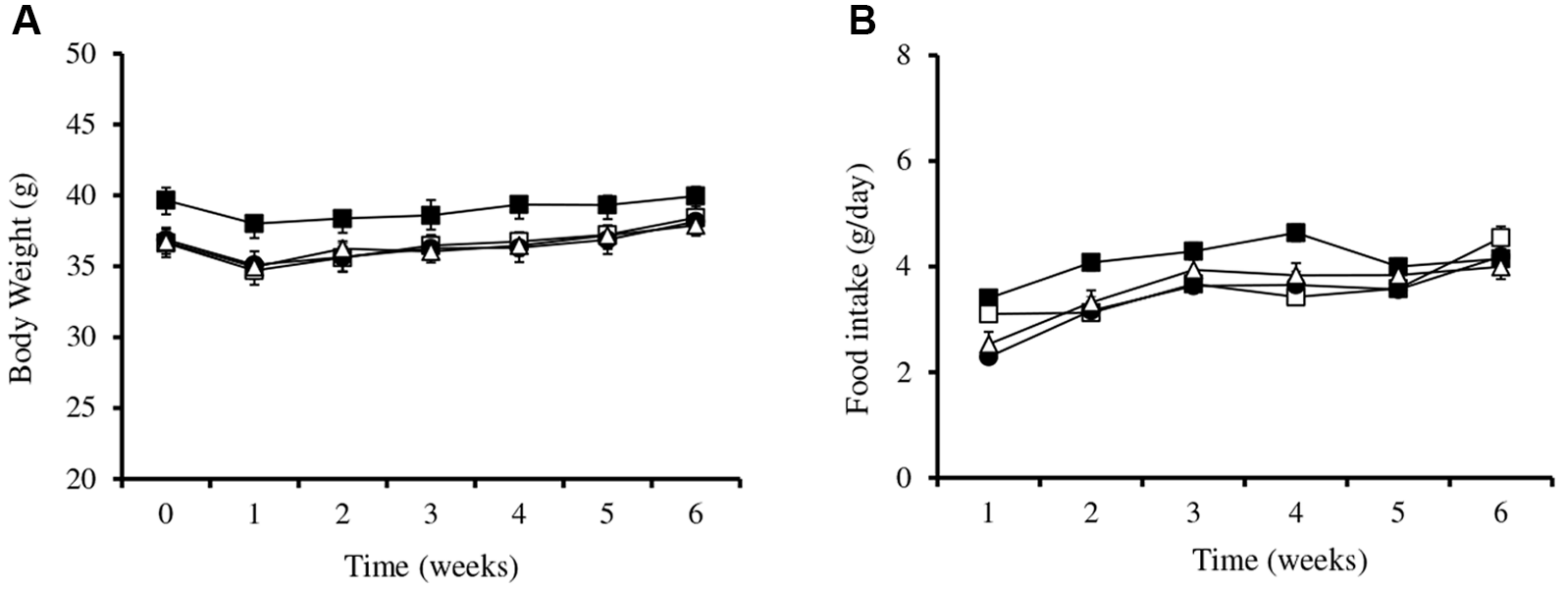
Bodyweight (A) and food intake (B) of ICR mice receiving various treatments. (■) Normal, (□) HCD, (●) *L.ac*, (Δ) KS6I1. The results are presented as mean ± SD (*n* = 6/group).

The bodyweight of mice and food intake of the control and treated groups over the period of 6 weeks are shown in Fig. 1. The bodyweight (Fig. 1A) and food intake (Fig. 1B) of the mice in HCD, KS6I1, *L.ac*, and Normal groups did not show significant difference.

**Fig. 2:**
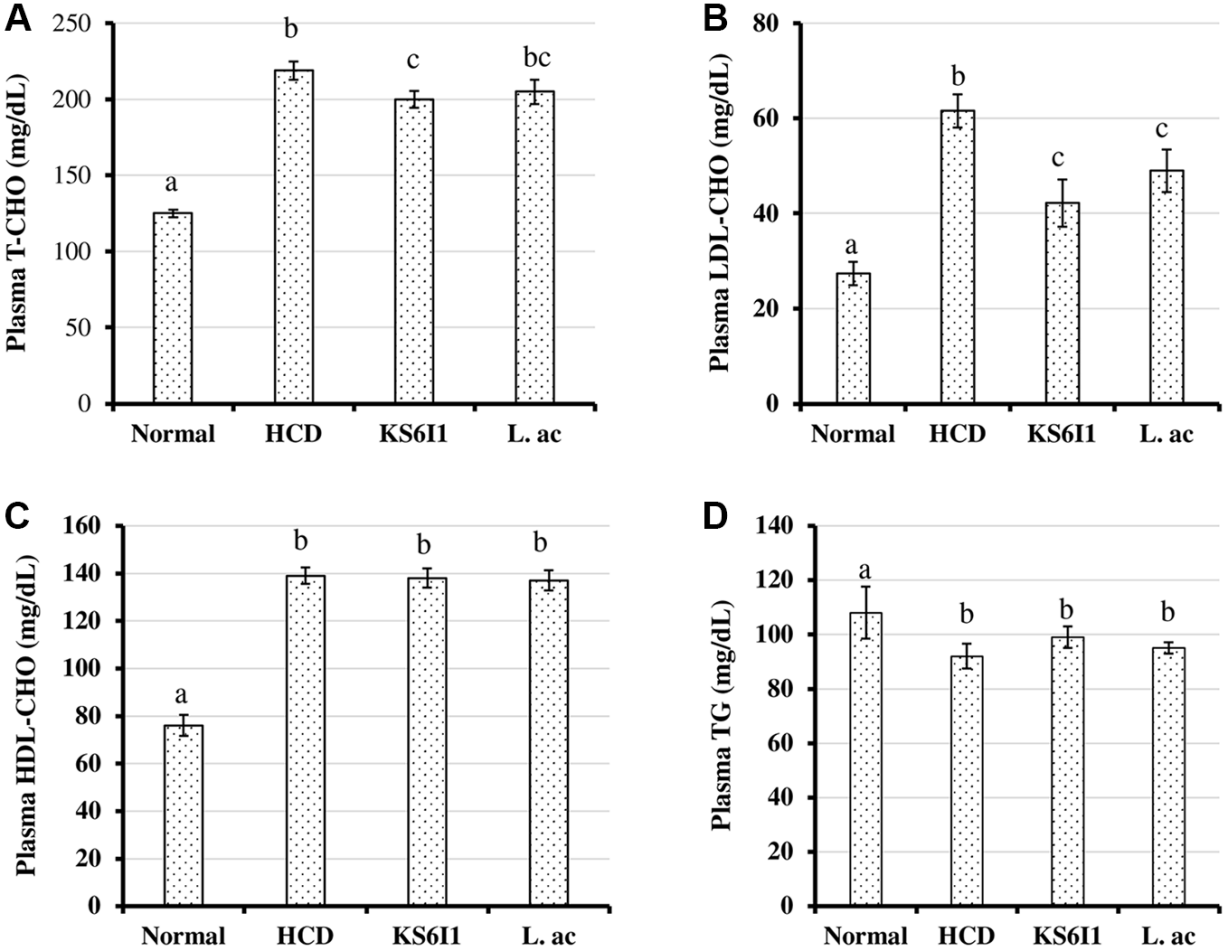
Effect of *L. pentosus* strain KS6I1 on plasma biochemical parameters such as total cholesterol (A) LDL cholesterol (B) HDL-cholesterol (C) and triglyceride (D) in mice after 6 weeks. Bars with different letters indicate significantly different from each other and bars sharing letters are not significantly different.

The levels of total cholesterol (Fig. 2A), HDL-cholesterol (Fig. 2C) and triglyceride (Fig. 2D) in plasma did not show any significant difference in mice belonging to the HCD and L.ac groups. However, the plasma LDL-cholesterol level was significantly decreased in the KS6I1 group and in L. ac group compared to the HCD group (*p* < 0.05) (Fig. 2B). KS6I1 group showed a significant reduction in total cholesterol compared to HCD.

### Effect of KS6I1 Feeding on Lipid Content in Liver Tissue

**Fig. 3:**
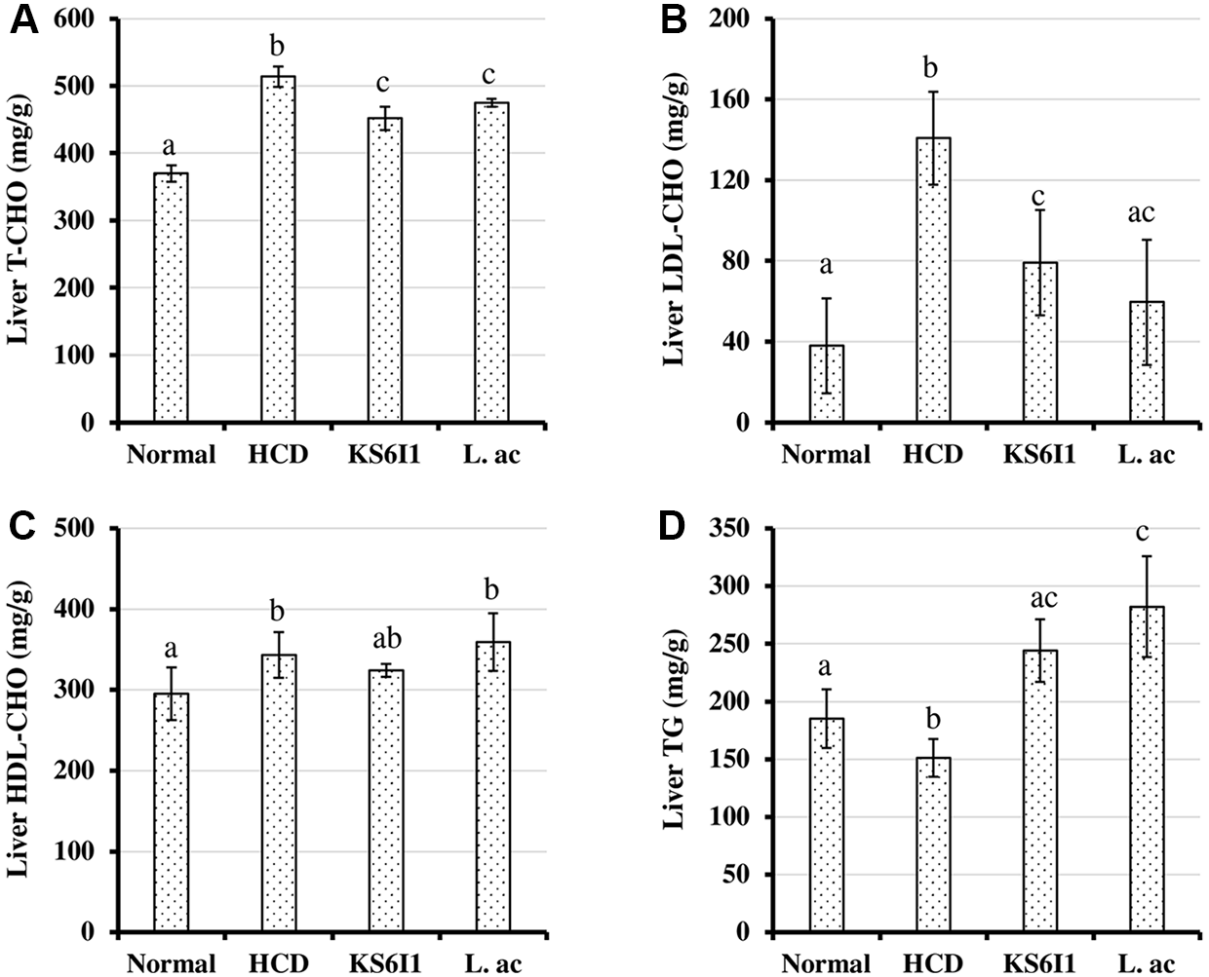
Effect of *L. pentosus* strain KS6I1 on liver biochemical parameters such as total cholesterol (A) LDL cholesterol (B) HDL-cholesterol (C) and triglyceride (D) in mice after 6 weeks. Bars with different letters indicate significantly different from each other and bars sharing letters are not significantly different.

The total cholesterol (Fig. 3A) and LDL-cholesterol (Fig. 3B) levels in liver tissue of mice belonging to KS6I1 and L.ac are significantly lower when compared to HCD group. There was no significant difference in the levels of HDL-cholesterol in the liver in mice belonging to HCD, KS6I1, and L.ac groups (Fig. 3C). The triglyceride levels in liver tissues in KS6I1 and L. ac groups were significantly elevated compared to HCD (Fig. 3D).

### Effect of KS6I1 Feeding on Lipid and Bile Acid Contents in Mice Feces

**Fig. 4:**
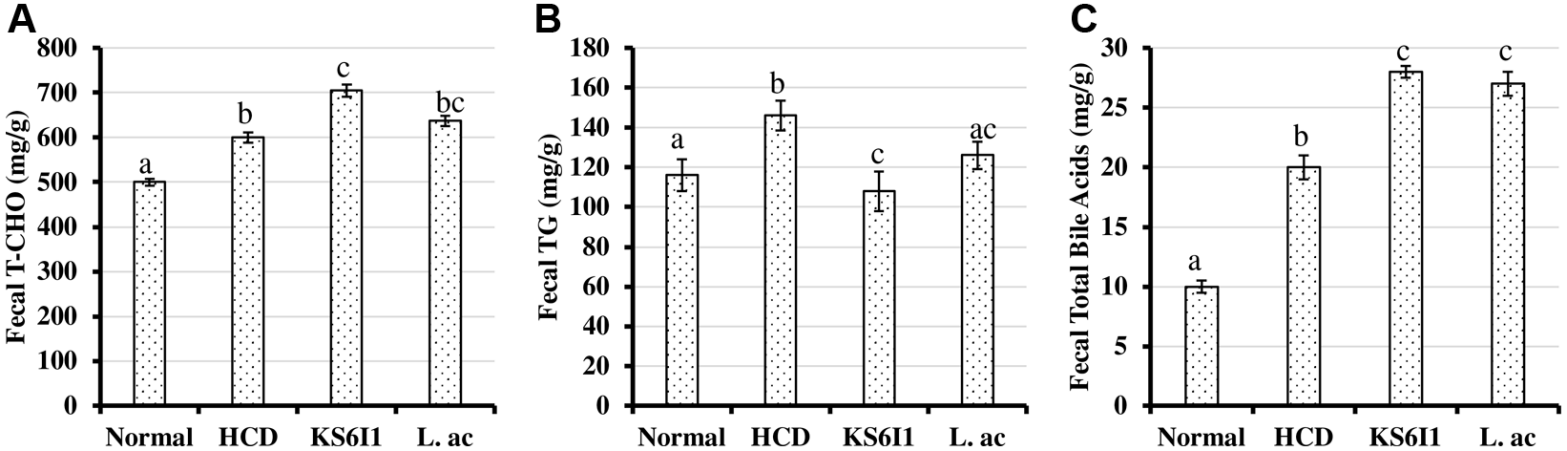
Effect of *L. pentosus* strain KS6I1 on fecal biochemical parameters such as total cholesterol (A) LDL cholesterol (B) HDL-cholesterol (C) triglyceride (D) and total bile acids (E) in mice after 6 weeks. Bars with different letters indicate significantly different from each other and bars sharing letters are not significantly different.

The total cholesterol levels in the feces were observed to be higher in KS6I1 and L.ac groups compared to that of Normal and HCD groups (Fig. 4A). The fecal triglyceride level (Fig. 4B) was significantly lower (*p* < 0.01) in KS6I1 than in other groups. Finally, the total bile acid content of the feces was significantly (*p* < 0.01) higher in the KS6I1 and L.ac group than in the NCD and HCD groups (Fig. 4C).

### Effects of KS6I1 on the Expression Levels of Genes Associated with Cholesterol Metabolism in the Liver

**Fig. 5:**
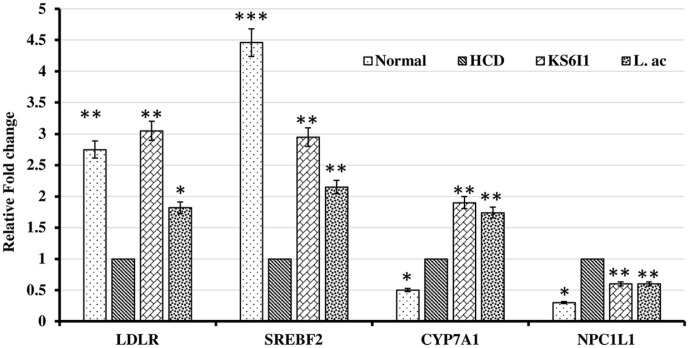
Effects of KS6I1 feeding on expression levels of cholesterol metabolism-related genes (LDL receptor, SREBP-2, and CYP7A1) in mice liver and NPC1L1 in mice intestine. Data were normalized to β-actin RNA expression levels and then compared to the HCD group. *, *p* < 0.05, **, *p* < 0.005, ***, *p* < 0.001, vs HCD group.

The expression of *LDLR* (LDL receptor) in the liver was higher in the KS6I1 and L.ac groups than in the HCD group (Fig. 5). A significant difference in the expression levels of *SREBF2* and *CYP7A1* genes were observed between the KS6I1, L.ac group and HCD groups. Additionally, the expression level of NPC1L1 gene is downregulated in KS6I1 and L.ac groups compared to HCD group (Fig. 5).

## Discussion

*L. pentosus* KS6I1 was evaluated for its in vivo cholesterol-lowering activity in hypercholesterolemic mice and compared with that of *L. acidophilus* ATCC 43121—a known cholesterol-lowering probiotic strain [53-59]. *L. pentosus* KS6I1 was previously reported to have in vitro cholesterol assimilation activity and showed a significant reduction in cholesterol level in MRS-CHO broth in the presence of 0.3% bile [47]. Additionally, *L. pentosus* KS6I1 also had bile salt hydrolase activity and could deconjugate taurocholic acid, glycocholic acid and taurodeoxycholic acid into taurine, glycine, cholic acid and deoxycholic acid [47]. Bile salt hydrolase activity is an important probiotic trait [32] and has been shown to play an important role in in vivo cholesterol-lowering activity [32, 48, 50]. Hence, we investigated the in vivo cholesterol-lowering activity of *L. pentosus* KS6I1 in high-cholesterol diet-induced hypercholesterolemic mice.

In this study, we observed no significant differences in the food intake and bodyweight gain of normal diet-fed mice and high cholesterol diet (HCD)-fed mice and HCD with lactic acid bacteria fed mice, which is similar to the results of our previous study using male ICR mice fed with high cholesterol diet plus *L. helveticus* strain KII13 [31] and high cholesterol diet plus *L. fermentum* MJM60397, indicating that feeding of lactobacillus strains does not affect food consumption or body weight.

Feeding of *L. pentosus* strain KS6I1 to hypercholesterolemic mice resulted in a significant decrease in plasma LDL-cholesterol and plasma total cholesterol levels compared to HCD-control and no significant change in HDL-cholesterol and triglyceride levels. A previous study showed that feeding *L. pentosus* KF923750 to hypercholesterolemic rabbits resulted in significant reduction in the total cholesterol, LDL-cholesterol and triglycerides with no significant change in HDL cholesterol levels in the blood plasma of the treated rabbits. Additionally, histological sections of livers from these rabbits showed less pronounced lesions in rabbits ingesting *L. pentosus* KF923750 compared to control [30]. A similar study reported that administration of *L. plantarum* KCTC3928 to C57BL/6 mice fed a high-fat diet exhibited a significant reduction in plasma LDL-C and triacylglycerol by 42% and 32%, respectively and increased the fecal bile excretion by 45% [39].

Analysis of lipid contents in the liver tissues of mice fed with KS6I1 showed a reduction in total cholesterol and LDL-cholesterol levels and the levels of triglycerides are significantly elevated (Fig. 3). Triglycerides are the major form of fatty acids storage and transport. Fatty acid metabolism takes place in the liver and accumulation of fatty acids takes place by hepatocellular uptake from the plasma and by *de novo* biosynthesis. Fatty acids are eliminated by β-oxidation within the cell or by secretion into the plasma within triglyceride-rich very low-density lipoproteins [60]. Alterations in the hepatic fatty acid metabolism commonly leads to increased absorption of triglycerides by liver [60]. Feeding of LAB could possibly alter fatty acid metabolism which is responsible for the observed increase in liver triglyceride levels (Fig. 3D). Also, we observed higher excretion of cholesterol and bile acids in the hypercholesterolemic mice fed with *L. pentosus* KS6I1 than in other groups (Fig. 4). These results indicate that *L. pentosus* KS6I1 is causing increased excretion of cholesterol and bile acids, which results in increased absorption of cholesterol by liver from the plasma indicated by decreased LDL-cholesterol in plasma (Fig. 2B).

Analysis of the gene expression related to cholesterol metabolism and absorption indicated that an increased expression of LDLR, SREBF2 and CYP7A1 in liver in KS6I1 and L.ac groups compared to HCD (Fig. 5). However, upregulation of the expression of the LDL receptor (LDLR) gene does not always lead to an increase in the LDLR protein [61]. The *LDLR* gene is regulated by the *SREBF-2*, upregulation of which inhibit liver absorption of cholesterol [62]. Another observation is the increased expression of CYP7A1 gene in KS6I1 and in L.ac, which leads to increased conversion of cholesterol into bile acids [62]. Furthermore, the levels of NPC1L1 gene are downregulated in the KS6I1 and L.ac groups compared to HCD. NPC1L1 gene is important for cholesterol absorption in the intestine. Downregulation of NPC1L1 gene expression in the intestine leads to inhibit the intestinal absorption of cholesterol [62]. Cholesterol is the precursor of bile acids and a decrease in bile acids in the body due to increased excretion will lead to utilization of cholesterol for synthesis of bile acids by the liver [32]. The higher expression of CYP7A1 gene in liver explains that cholesterol is being converted to bile acids.

Analysis of gene expression in cholesterol-lowering activity differed among different lactic acid bacteria reported earlier. *L. plantarum* KCTC3928 with cholesterol-lowering activity was reported to alter the expression levels of LDLR and HMGCR genes marginally and significantly elevate the expression level of CYP7A1 gene in mice [39]. Additionally, probiotic *Lactobacillus acidophilus* ATCC 4356 reduced NPC1L1 gene expression and inhibited the cellular uptake of micellar cholesterol in Caco-2 cells [63]. Soluble effector molecules secreted by ATCC 4356 were reported to cause the decreased expression of NPC1L1 [63].

KS6I1 strain exhibits multiple cholesterol-lowering mechanisms such as it lowers cholesterol by decreasing bile acid absorption by its bile salt deconjugating activity, which results in increased fecal excretion of bile acids. Deconjugated bile acids are poorly reabsorbed than conjugated bile acids and results in the excretion of larger amounts of free bile acids in feces. Additionally, free bile acids are poor in solubilizing lipids, which leads to reduced lipid absorption in the intestine [32]. Bile salt hydrolase activity in the gut will lead to a reduction in plasma cholesterol either by increasing *de novo* synthesis of bile acids or by reducing cholesterol solubility and absorption in the intestine [32, 50]. Furthermore, KS6I1 increases excretion of cholesterol in the feces due in part to its cholesterol assimilation activity reported earlier [47] and downregulating the expression of NPC1L1 gene in the intestine. In conclusion, *L. pentosus* KS6I1 is a promising probiotic strain with in vivo cholesterol-lowering activity by increasing the fecal excretion of bile acid and cholesterol in mice.
